# Recent Trends in Injury Models to Study Skeletal Muscle Regeneration and Repair

**DOI:** 10.3390/bioengineering7030076

**Published:** 2020-07-20

**Authors:** Sydnee T. Sicherer, Rashmi S. Venkatarama, Jonathan M. Grasman

**Affiliations:** Department of Biomedical Engineering, New Jersey Institute of Technology, Newark, NJ 07102, USA; sts27@njit.edu (S.T.S.); rsv24@njit.edu (R.S.V.)

**Keywords:** skeletal muscle regeneration, tissue engineering, volumetric muscle loss, animal models

## Abstract

Skeletal muscle injuries that occur from traumatic incidents, such as those caused by car accidents or surgical resections, or from injuries sustained on the battlefield, result in the loss of functionality of the injured muscle. To understand skeletal muscle regeneration and to better treat these large scale injuries, termed volumetric muscle loss (VML), in vivo injury models exploring the innate mechanisms of muscle injury and repair are essential for the creation of clinically applicable treatments. While the end result of a muscle injury is often the destruction of muscle tissue, the manner in which these injuries are induced as well as the response from the innate repair mechanisms found in muscle in each animal models can vary. This targeted review describes injury models that assess both skeletal muscle regeneration (i.e., the response of muscle to myotoxin or ischemic injury) and skeletal muscle repair (i.e., VML injury). We aimed to summarize the injury models used in the field of skeletal muscle tissue engineering, paying particular attention to strategies to induce muscle damage and how to standardize injury conditions for future experiments.

## 1. Introduction

**Figure 1:**
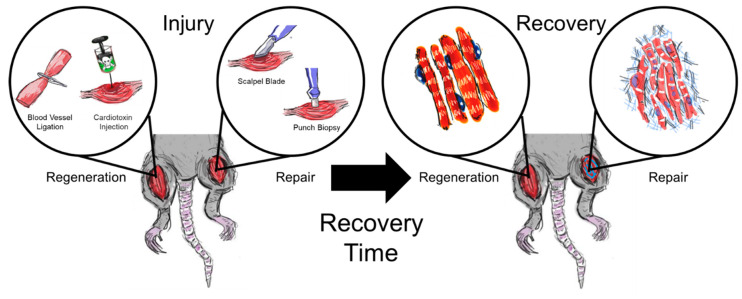
Injury models used to assess skeletal muscle regeneration and repair. Injury induction for regenerative models (“regeneration”) regenerate with high fidelity after recovery, while repair models (“repair”) result in some nascent myofibers interspersed with fibrotic tissue.

Skeletal muscle is responsible for the coordinated locomotion of the skeleton, resulting in ambulation of the organism. This highly aligned organ has an innate repair mechanism, which supports day-to-day function and facilitates recovery from small injuries (for details, see [1,2]). However, larger injuries, classified as volumetric muscle loss (VML), overwhelm this innate repair mechanism and result in the complete removal of at least 20% of muscle by mass, leading to dysfunctional and incomplete functional repair [1]. Skeletal muscle is comprised of several tissues, namely, myofibers, blood vessels, nerves, and connective tissue, making it a complex organ that is not easily regenerated in situ after traumatic injury. Inadequate regeneration will lead to inefficient transmission of force, which will result in loss of function and a reduction in the patient’s quality of life. Investigators have developed a variety of scaffold and cell-based interventions to advance skeletal muscle tissue engineering and address this growing need. While a systematic review of these numerous strategies is outside the scope of this review (for an overview, see [2,3,4,5,6]), a common question is how to assess the applicability of these strategies. To this end, a variety of animal models have been developed to study skeletal muscle repair and regeneration (Figure 1).

In this review, we critically assess recent injury models designed to study muscle regeneration (e.g., myotoxins or ischemia), as these methods have identified mechanistic steps important for skeletal muscle regeneration and are incredibly useful to determine the effects of cells or soluble factors on muscle regeneration (Figure 1, regeneration). We next focus on VML injury models, which assess skeletal muscle repair after traumatic injury (Figure 1, repair). We distinguish repair from regeneration, because while myotoxin injuries can regenerate with high fidelity to their original, uninjured state, VML injuries cannot spontaneously regenerate, and to date clinical interventions have not resulted in complete functional recovery [7]. Therefore, repair in this case refers to our ability to augment skeletal muscle force production, often by mimicking processes found in regeneration. While many of these methods are similar, there can be small differences in protocols that make comparison between studies difficult. We will summarize advantages of each of these methodologies, and discuss the need to establish common injury procedures, particularly for VML injury models to maximize comparisons between studies to be able to ultimately translate tissue engineered strategies for skeletal muscle regeneration into the clinic.

## 2. Myotoxins

Myotoxins are a family of proteins commonly found in snake venoms that result in massive amounts of myolysis and necrosis. Cardiotoxin (CTX), a specific myotoxin derived from cobras, renders the cell membrane more permeable to ion influx, destroying large regions of the muscle and significantly impacting functional outputs [8]. Myotoxin-induced injury models are typically utilized to study regeneration because while the toxin completely destroys the muscle fibers, it leaves the extracellular matrix (ECM) intact and does not specifically target satellite cells, allowing them to regenerate the tissue [9,10,11]. The muscle is able to recover most of its original contractile parameters (maximum tetanic force, contraction time, and maximum power) 10 days after injury, and after 3 weeks there are no differences in the contractile properties [9]. CTX is typically injected directly into the belly of a muscle, such as the tibialis anterior (TA). This intramuscular injection is a relatively simple procedure, resulting in a reproducible and consistent injury model [12]. Further, the relatively small size of the TA ensures a greater portion of the muscle will be damaged by the toxin, generating a severe injury [13]. The combination of a fast recovery time with acute, localized damage makes myotoxin models ideal to answer mechanistic molecular questions regarding skeletal muscle regeneration (Table 1). For example, the role of glycans, such as Galgt1, in skeletal muscle regeneration was identified by comparing regeneration in normal and Galgt1-deficient mice after CTX injury [14]. Despite observing regeneration in both models, the myofiber diameter in Galgt1-deficient mice was significantly smaller 2 weeks after recovery, demonstrating the importance of Galgt1 in regeneration and that it may be targeted when there is insufficient or slow regeneration [14]. In addition to cell surface receptors, this platform allows for the study of soluble factors, such as angiopoietin-1 (Ang-1). Fourteen days after CTX injection into the TA muscle, exogenous Ang-1 significantly increased muscle contractility, fiber regeneration, and capillary density [15]. Anti-inflammatory compounds such as thymol were found to accelerate skeletal muscle regeneration by observing a reduction in the number of mast cells and a decrease in the final percent of collagen after CTX injection into the gastrocnemius muscle [16]. However, while myotoxin injection is an ideal model to understand the underlying mechanisms of skeletal muscle regeneration, it is unable to model the frank loss of tissue that occurs in traumatic injury, such as those presenting from VML, limiting its relevance for clinical injuries.

## 3. Ischemia

Ischemia is a result of loss of blood flow and oxygen in the body. Ischemia models, specifically those that involve a permanent ligation of vessels leading to muscles, result in muscle loss and relatively low levels of spontaneous functional recovery compared to other injury models [18]. Borselli et al. compared muscle recovery following a permanent ligation of the TA and gracilis muscles with and without growth factor-loaded hydrogels to show that delivery of exogenous vascular endothelial growth factor (VEGF) and insulin-like growth factor 1 (IGF1) accelerated muscle recovery (Table 2) [18]. This recovery was observed by measuring significant increases in myofiber diameter as well as tetanic force with respect to hydrogels without VEGF or IGF1, 2 and 7 weeks after injury [18]. In a similar study, delivery of exogenous VEGF was shown to improve muscle recovery after ischemia by delaying axonal degeneration and increasing proliferation of neural progenitor cells, thereby maintaining muscle innervation [19]. Ischemia/reperfusion (IR) injury models differ from permanent ligation in that blood flow is reestablished after several hours of acute ischemia, which can result in swelling, destruction of the capillary network, and even organ failure [20,21]. Skeletal muscle is particularly vulnerable to IR injury, and efforts to mitigate tissue damage include the injection of bone marrow cells (BMCs) into mice 2 days after IR injury. An improvement in maximal tetanic torque was observed 16 days post-injury, as well as a faster rate of force production and a decrease in fibers with centrally located nuclei, demonstrating that injected BMCs aided regeneration [20]. Another study injected exogenous sonic hedgehog (Shh) to the hindlimb at the onset of ischemia and observed that the delivered Shh enhanced recovery, reduced fibrosis, and inhibited apoptosis [22]. IR models are not as widely used to study muscle regeneration because they are less efficient at muscle recovery than injection of myotoxins [23]. For example, there was delayed fatigue resistance and muscle weight in the IR model when compared to myotoxic models, even though the muscle fibers were initially completely destroyed in both [23]. Despite these limitations, IR injuries can mimic surgical transfers of muscle, which is of significance for tissue transfer techniques for traumatic injuries.

## 4. VML Injury Models

Increased attention in recent years has been focused on exploring treatment options for volumetric muscle loss (VML) and as such there have been numerous efforts to characterize and define animal models. However, there is a large amount of variation in the experimental setup between each study, including animal species/strain, the location of VML injury, and the extent of the VML injury induced. We briefly summarize animal models used for VML (Table 3), highlighting characteristics that could be conserved between studies to properly allow for comparisons between multiple studies (for a comparison of studies using meta-data analysis, see [25]).

### 4.1. Animal Species Used in VML Studies

The most common animals employed in VML studies are rodents (mice and rats), although larger mammals such as sheep, canines, and pigs have also been studied due to their clinically relevant size [36,37]. Approximately 88% of studies examining decellularized extracellular matrix (ECM) implant materials on VML injuries utilized mouse or rat models, while only 12% utilized larger animal models such as sheep, dogs, and pigs [37]. Generally, variations in animal strains can be accredited to the nature of the construct being studied. In studies utilizing xenogeneic cells, immunodeficient animals are required to mitigate transplant rejection [26,28,38]. Human cell sources are commonly used to maximize the translational impact, focusing on the integration of skeletal muscle mimetics into the host. In this way, Page et al. implanted human myoblast-seeded fibrin microthreads into the tibialis anterior (TA) muscle of mice, observing functional recovery as well as human myoblast engraftment with host tissue [39]. Scaffolds seeded with human myogenic stem cells (HMuSCs) and human muscle resident cells (HMRCs; non-myogenic muscle cells) were implanted into the TA, resulting in new muscle formation and minimal scarring. Importantly, scaffolds seeded with either human-sourced or autologous (mouse) cells resulted in similar recovery, highlighting the translational feasibility assessing engraftment into host tissues [28]. Separately, to dissect the contribution of immune cells in the repair of VML injuries, minced autologous muscle tissue was implanted into wild-type and athymic mice [26]. In this way, it was demonstrated that immune cells play an important role in satellite cell proliferation in VML injuries (for a comprehensive review, see [40]) and that the overall modulation of the immune response may be necessary [26]. While immunodeficient animals are necessary to study the integration and repair of xenogeneic cell-seeded scaffolds, these animal models alone cannot provide insight into how cells, scaffolds, and host tissues will integrate into a fully functioning biological system.

Immunocompetent mouse and rat models are used in studies where the risk of a systemic adaptive immune response is reduced, as well as to increase clinical relevance. Bladder acellular matrices (BAMs) seeded with allogenic muscle progenitor cells (MPCs), called tissue-engineered muscle repair (TEMR), elicited no adverse immune reactions in Lewis rats who responded positively to the implant [29]. The presence of macrophages 24 weeks post injury within the VML injury site indicated that, while an immune response did occur, it had stabilized and was conducive to muscle repair and regeneration [29]. Another treatment option under investigation is the implantation of decellularized scaffolds seeded with autologous minced muscle tissue. Implanting these materials into the TA of Fischer 344 rats improved muscle mass and reduced fibrosis without any notable inflammatory response [31].

While mouse and rat models provide insight into evaluating repair mechanisms for the treatment of VML injuries, their clinical relevance is limited by the small size of both the muscles and the injuries. These injuries do not accurately recapitulate the clinical scenario where defects are in the order of cubic inches rather than cubic millimeters, approximately 1000× times larger. For example, the implantation of skeletal muscle units (SMUs) into a rat TA VML injury resulted in improved force production, innervation, and vascularization throughout the construct [30]. To translate these findings towards clinically relevant injuries, researchers implanted allogeneic SMUs into a VML injury in the peroneus tertius muscle of sheep. Sheep implanted with SMUs and engineered neural conduits (ENC; SMU+ENC) maintained increased muscle mass and increased muscle contraction 3 months post injury compared to sheep who did not receive treatment [36]. While the results of this study demonstrated that implantation of SMU+ENC scaffolds improved VML recovery, the extent of force recovery was less than that observed in the prior rat studies, possibly a result of the increased reliance on the diffusion of nutrients into the implanted construct [36]. In a similar series of studies, minced autologous muscle tissue was implanted into a VML defect in the peroneus tertius muscle of Yorkshire-cross pigs, after initial results in rat models showed that the implantation of autologous minced tissue resulted in improved functional recovery and muscle regeneration [26]. While autologous tissue implants restored 32% of strength lost from the VML injury, the size of the defect limited the repair capabilities in pigs [35]. Despite the positive results observed in both rat VML models, the relative amount of functional recovery and regeneration was reduced in larger models.

With the majority of VML studies utilizing mice and rats, the utilization of larger animals could overcome a significant barrier in the design and interpretation of VML studies. While clearly biologically more relevant, there are relatively few large animal models, making comparisons to related studies difficult. The usage of larger animal models is also limited by the price, facilities, and materials required to care for them. In this way, pioneering new animal models is limited by both the lack of studies, as well as the increased cost required. Although large animals may be more biologically relevant to human VML injuries, the requirements to study such do not always make them a viable option, particularly for smaller laboratories.

### 4.2. Variations in Muscle Group

VML injuries have been induced in a variety of muscle groups including the TA [28,41], quadriceps femoris [33,34], and latissimus dorsi (LD) [42,43]. The most commonly used muscle in VML studies is the TA, most likely due to the fact that it is easy to access and the metrics for testing its functional capabilities have been well established. In all studies, the main assessment of muscle repair is functional recovery via measuring torque production by hindlimbs with and without VML injuries [41]. While functional recovery remains the principle assessment, the methodologies can change because of both the differences in the anatomical structure and location of these muscles. In the LD, functional measurements were evaluated by removing the entire muscle and measuring its contractile force in vitro [43] or by keeping the muscle intact and measuring the isometric force in situ by affixing the insertion tendon to a load cell [42]. Functional recovery of the quadriceps femoris was also determined by measuring the tetanic torque of the limb in situ but was also determined by measuring changes in the gait of the rats over several weeks [33]. While functional recovery was observed in each of these studies, the extent to which recovery occurred in the quadriceps femoris was less than that of the other two muscle groups, potentially due to collateral injury of the nerves surrounding the muscle [33].

While all of these muscles are composed of the same cellular components, the variation in muscle organization and function presents different design considerations for researchers. For instance, the TA is fusiform and circumpennate; the LD is unipennate; and the rectus femoris, one of the muscles in the quadriceps, is bipennate, which will impact fiber organization and force transmission [44,45]. These different structural characteristics highlight an underlying difference in myofiber organization—circumpennate muscles are cylindrical and converge to a central tendon, unipennate muscles extend outward from one side of a tendon in a feather-like pattern, and bipennate muscles are similar to unipennate muscles but have myofibers connecting to both sides of the tendon. In many cases, researchers attempt to maximize linear alignment of myofibers, under the assumption that all muscles are fusiform or unipennate, and therefore do not adjust for variations in myofiber alignment for different anatomies. Ultimately, while studies may utilize similar materials and methods to repair VML injuries, the anatomy and orientation of the muscle fibers may impact the degree of recovery, confounding results and comparisons between studies utilizing different muscle groups.

### 4.3. Size and Induction of Injury

By definition, VML is induced by the removal of muscle tissue; however, researchers achieve such via a variety of different tools and procedures. The removal of 40% of the TA resulted in a 40% loss in functional strength compared to controls [28], while a study utilizing the same model found that the removal of 30–50% of the TA muscle resulted in a 30% loss in functional strength [46]. Small variations in tissue removal can impact functional outcomes, and it is important to note that there is no universal linear relationship between tissue removal and force reduction; these parameters are based on many factors such as the geometry of the injury and the muscle anatomy [32]. In some studies, as little as 20% of the TA muscle was removed [47], while in others as much as 75% of the quadriceps compartment was removed [48]. Some studies report the removal of defined volumes, such as the removal of 4 × 2 × 2 mm^3^ of tissue in the mouse TA using iris scissors and wedge resection [27,38], or a 2 mm biopsy punch in the same muscle group [26]. The use of a stencil allows for repeatable injuries, as well as assisting readers in understanding the size of the injury [28]; however, some studies do not clearly define these geometries. An augmentation to this strategy has been to remove a specific dimension of muscle and report the functional reduction of the muscle group as the final metric for injury induction [27]. An alternative strategy removes 20% by mass from the middle third of the TA on the basis of a linear-regression model that determines the mass of the muscle removed in relation to the total body weight of the animal [49]. While all these approaches report a certain percentage of muscle removal, the method of removal may likely result in inconsistencies between the actual percent of muscle removed, therefore making cross-study comparisons difficult [25]. While there does not appear to be significant variation in the results on the basis of these varying methodologies, the practice of removing defined amounts (i.e., length × width × height measurements) of muscle tissue to induce VML injury seems to be a more preferred method.

In addition to variations in the amount of removed muscle mass, the treatment of other muscles in the same compartment can impact the injury model. Several studies mechanically isolate the TA from the other anterior crural muscles to prevent these muscles from compensating for the injury [49]. A separate approach has been to leave these other muscles intact, instead directly stimulating the surface of the muscle of interest [27,48]. While this approach does not induce injuries of multiple muscles, it requires additional controls such as age-matched animals to ensure that muscle compensation is not occurring. The aim of ablating muscles in a similar compartment to the muscle of interest (e.g., the TA and the other anterior crural muscles) is to eliminate the possibility of the other muscles to strengthen and compensate for the injured muscle via hypertrophy. Quarta et al. described several studies that demonstrated that the effects of this hypertrophic mechanism are minimal in mice, even when the muscles surrounding the TA are not removed [28]. Ultimately, the ablation of muscles in the same compartment may not be necessary; however, care must be taken to confirm this in the experimental design by utilizing additional controls to confirm that there is no hypertrophy of the muscles around the injured muscle.

## 5. Summary and Future Considerations

Many injury models have been developed to answer fundamental questions in the field, ranging from mechanistic processes underlying regeneration to the repair of traumatic injuries. The use of regenerative injury models, such as myotoxins, should be reserved for mechanistic questions regarding skeletal muscle regeneration, as they destroy muscle fibers while leaving the ECM intact. Ischemia/reperfusion models may be relevant to enhance the survival of tissue transfer techniques. VML injuries are relevant to assess skeletal muscle repair, which involves the frank loss of tissue. To enable more direct comparisons between VML studies, we make the following recommendations as investigators continue to innovate and improve scaffold- and/or cell-based interventions: (1) the presentation of as much information as possible regarding the induction of the VML injury (i.e., wound dimensions, mass of tissue loss, and reduction of force output) would greatly increase both the repeatability of each injury model, as well as comparing newer studies with existing literature. Where possible, each axis of the wound dimensions should be defined (e.g., length × width × height), and should provide information regarding the origin of the injury, as many muscle geometries will be impacted by the location of the injury. (2) Forces should be recorded before and after injury, with an option to utilize reduction of force output as an additional metric for injury induction. (3) Utilization of immunocompetent animals where possible to maximize clinical relevance of the immune system. Of course, where there is an overt risk of a systemic adaptive immune response, such as the use of human cells within rodents, immunodeficient animals are necessary. (4) While a cross-comparison between two specific strains of mice did not reveal differences in functional recovery after injury [26], care and conservation in the selection of animal strains is necessary to ensure molecular differences between strains do not confound results between studies. (5) Investigators can choose to measure functional recovery via force recordings in vivo, in situ, or in vitro; however, care needs to be taken to clearly articulate the selected force collection method. Where appropriate, additional controls to account for hypertrophy and compensation from other muscles within the same compartment is necessary. (6) Small animals remain the primary vehicle to assess VML repair, and while it is important to consider the repair of different muscle groups, care needs to be taken to account for differences in myofiber organization, which presents unique engineering challenges to account for differences in anatomy between muscles. For example, the TA, the LD, and the quadriceps each have distinct geometries, and therefore comparison between them will be difficult, at best. (7) Scaling up treatment options from these smaller injuries to clinically sized defects remains one of the most significant challenges in the field, despite the many innovative advances in skeletal muscle tissue engineering to date.

## Figures and Tables

**Table 1:** Summary of recent studies utilizing cardiotoxin (CTX) injuries to study skeletal muscle tissue regeneration.

Injury Model	Treatment	Results	Reference
Mice CTX gastrocnemius muscle	Thymol	Significant decrease in the number of mast cells and the final percent of collagen after 10 days.	[16]
Mice CTX TA muscle	Ang-1	Ang-1 increases muscle contractility, fiber regeneration, and capillary density after 14 days.	[15]
Mice CTX TA muscle	Galgt1	Galgt1 expression resulted in larger myofiber diameters and more markers of muscle regeneration after 2–4 weeks.	[14]
Mice CTX TA muscle	Glycosaminoglycan mimetics	Injection of mimetics increased nuclei per myofiber and enhanced capillary formation within muscles.	[17]

Abbreviations: CTX (cardiotoxin), TA (tibialis anterior), Ang-1 (angiopoietin 1).

**Table 2:** Recent studies utilizing ischemia (ligation and ischemia/reperfusion (IR)) to study skeletal muscle regeneration.

Injury Model	Treatment	Results	Reference
Mice Ischemia ligation Hindlimb	VEGF and IGF1	Improvement in vasculature as well as muscle contractility and myofiber diameter after 7 weeks.	[18]
Mice Ischemia ligation TA muscle	VEGF	Enhanced vascularization of muscle, leading to expression of neurotrophic factors to maintain innervation.	[19]
Mice IR Upper hindlimb	Bone marrow cells	After 4 weeks, there was an increase in maximal tetanic torque and force production and a decrease in centrally located nuclei.	[20]
Rats IR Upper hindlimb	Vagus nerve stimulation	Stimulation led to reduction in apoptosis and inflammation, as well as protection of vascular endothelial function.	[24]
Mice IR Hindlimb	Shh	Improved myofiber recovery, inhibited apoptosis, and reduced fibrosis.	[22]

Abbreviations: VEGF (vascular endothelial growth factor), IGF1 (insulin-like growth factor 1), TA (tibialis anterior), IR (ischemia/reperfusion), Shh (sonic hedgehog).

**Table 3:** Selection of recent volumetric muscle loss (VML) studies to study the skeletal muscle repair.

Animal Model	Location	Injury Size	Treatment	Results	Reference
IC and ID mice	TA	2 mm biopsy punch (20% by mass)	Minced muscle from GFP+ mice	Muscle repair response was similar in both strains despite no improvement in TA muscle strength.	[26]
ID mice	TA	4 × 2 × 2 mm^3^ (50% reduction in force)	Crosslinked fibrin microthreads loaded with HGF	Recovery of 200% of force production and sustained angiogenesis after 2 months.	[27]
ID mice	TA	2 × 7 × 2 mm^3^ (40% by mass)	Human MuSC^+^/MRC^+^ constructs	Increase in functional recovery with exercise and improved vascularization and innervation after 1 month.	[28]
IC rats	TA	10 × 5 × 7 mm^3^ (20% by mass)	BAM or TEMR	Significant functional recovery in TEMR responders, with mature muscle found in injury site after 6 months.	[29]
IC rats	TA	30% by volume (longitudinal cut)	SMUs	Significant functional recovery with evidence of nerve and blood vessel infiltration within SMU after 1 month.	[30]
IC rats	TA	8 × 3 mm^2^ deep biopsy punch (20% by mass)	Muscle-derived ECM with minced muscle	Significant functional recovery and reduced fibrotic response.	[31]
IC rats	LD	1.5 × 1.1 cm^2^ (13% by mass)	BAM or TEMR	Recovery of 71% of force production; enhanced angiogenesis after 2 months.	[32]
IC rats	Q	8 mm biopsy punch	Muscle autograft	No significant recovery of muscle function.	[33]
IC mice	Q	2, 3, or 4 mm biopsy punch, full thickness (5%, 15%, or 30% by mass)	No treatment	Threshold for VML defect was 3 mm biopsy punch.	[34]
Yorkshire-cross pigs	PT	3 × 3 × 1.5 cm^3^ (20% by mass)	Autologous minced muscle	32% strength increase after 4 months; extensive fibrotic tissue deposition.	[35]
Polypay sheep	PT	30% by volume (longitudinal cut)	SMUs + ENCs	Implants significantly increased force production after 3 months.	[36]

Abbreviations: IC (immunocompetent), ID (immunodeficient), TA (tibialis anterior), LD (latissimus dorsi), Q (quadriceps), PT (peroneous tertius), GFP (green fluorescent protein), HGF (hepatocyte growth factor), MuSC (muscle stem cell), MRC (muscle resident cell), BAM (bladder acellular matrix), TEMR (tissue-engineered muscle repair), SMU (skeletal muscle unit), ECM (extracellular matrix), ENC (engineered neural conduit), VML (volumetric muscle loss).

## References

[1] Grogan B.F., Hsu J.R., Skeletal C. (2011). Trauma Research, Volumetric muscle loss. J. Am. Acad. Orthop. Surg..

[2] Grasman J.M., Zayas M.J., Page R.L., Pins G.D. (2015). Biomimetic scaffolds for regeneration of volumetric muscle loss in skeletal muscle injuries. Acta Biomater..

[3] Pantelic M.N., Larkin L.M. (2018). Stem Cells for Skeletal Muscle Tissue Engineering. Tissue Eng. Part B Rev..

[4] Gilbert-Honick J., Grayson W. (2020). Vascularized and Innervated Skeletal Muscle Tissue Engineering. Adv. Healthc. Mater..

[5] Gholobova D., Terrie L., Gerard M., Declercq H., Thorrez L. (2020). Vascularization of tissue-engineered skeletal muscle constructs. Biomaterials.

[6] Dunn A., Talovic M., Patel K., Patel A., Marcinczyk M., Garg K. (2019). Biomaterial and stem cell-based strategies for skeletal muscle regeneration. J. Orthop. Res..

[7] Dziki J., Badylak S., Yabroudi M., Sicari B., Ambrosio F., Stearns K., Turner N., Wyse A., Boninger M.L., Brown E.H.P. (2016). An acellular biologic scaffold treatment for volumetric muscle loss: Results of a 13-patient cohort study. NPJ Regen. Med..

[8] Lomonte B., Rangel J. (2012). Snake venom Lys49 myotoxins: From phospholipases A(2) to non-enzymatic membrane disruptors. Toxicon.

[9] Vignaud A., Cebrian J., Martelly I., Caruelle J.P., Ferry A. (2005). Effect of anti-inflammatory and antioxidant drugs on the long-term repair of severely injured mouse skeletal muscle. Exp. Physiol..

[10] Buono R., Vantaggiato C., Pisa V., Azzoni E., Bassi M.T., Brunelli S., Sciorati C., Clementi E. (2012). Nitric oxide sustains long-term skeletal muscle regeneration by regulating fate of satellite cells via signaling pathways requiring Vangl2 and cyclic GMP. Stem Cells.

[11] Juhas M., Abutaleb N., Wang J.T., Ye J., Shaikh Z., Sriworarat C., Qian Y., Bursac N. (2018). Incorporation of macrophages into engineered skeletal muscle enables enhanced muscle regeneration. Nat. Biomed. Eng..

[12] Garry G.A., Antony M.L., Garry D.J. (2016). Cardiotoxin Induced Injury and Skeletal Muscle Regeneration. Methods Mol. Biol..

[13] Foltz S.J., Modi J.N., Melick G.A., Abousaud M.I., Luan J., Fortunato M.J., Beedle A.M. (2016). Abnormal Skeletal Muscle Regeneration plus Mild Alterations in Mature Fiber Type Specification in Fktn-Deficient Dystroglycanopathy Muscular Dystrophy Mice. PLoS ONE.

[14] Singhal N., Martin P.T. (2015). A role for Galgt1 in skeletal muscle regeneration. Skelet. Muscle.

[15] Mofarrahi M., McClung J.M., Kontos C.D., Davis E.C., Tappuni B., Moroz N., Pickett A.E., Huck L., Harel S., Danialou G. (2015). Angiopoietin-1 enhances skeletal muscle regeneration in mice. Am. J. Physiol. Regul. Integr. Comp. Physiol..

[16] Cardoso E.S., Santana T.A., Diniz P.B., Montalvao M.M., Bani C.C., Thomazzi S.M. (2016). Thymol accelerates the recovery of the skeletal muscle of mice injured with cardiotoxin. J. Pharm. Pharmacol..

[17] Bouviere J., Trignol A., Hoang D.H., del Carmine P., Goriot M.E., Larbi S.B., Barritault D., Banzet S., Chazaud B. (2019). Heparan sulfate mimetic accelerate post-injury skeletal muscle regeneration. Tissue Eng. Part A.

[18] Borselli C., Storrie H., Benesch-Lee F., Shvartsman D., Cezar C., Lichtman J.W., Vandenburgh H.H., Mooney D.J. (2010). Functional muscle regeneration with combined delivery of angiogenesis and myogenesis factors. Proc. Natl. Acad. Sci. USA.

[19] Shvartsman D., Storrie-White H., Lee K., Kearney C., Brudno Y., Ho N., Cezar C., McCann C., Anderson E., Koullias J. (2014). Sustained delivery of VEGF maintains innervation and promotes reperfusion in ischemic skeletal muscles via NGF/GDNF signaling. Mol. Ther..

[20] Corona B.T., Rathbone C.R. (2014). Accelerated functional recovery after skeletal muscle ischemia-reperfusion injury using freshly isolated bone marrow cells. J. Surg. Res..

[21] Blaisdell F. (2002). The pathophysiology of skeletal muscle ischemia and the reperfusion syndrome: A review. Cardiovasc. Surg..

[22] Zeng Q., Fu Q., Wang X., Zhao Y., Liu H., Li Z., Li F. (2017). Protective Effects of Sonic Hedgehog Against Ischemia/Reperfusion Injury in Mouse Skeletal Muscle via AKT/mTOR/p70S6K Signaling. Cell. Physiol. Biochem..

[23] Vignaud A., Hourde C., Medja F., Agbulut O., Butler-Browne G., Ferry A. (2010). Impaired skeletal muscle repair after ischemia-reperfusion injury in mice. J. Biomed. Biotechnol..

[24] Zhang Y., Li H., Wang M., Meng G., Wang Z., Deng J., Wang M., Zhang Q., Yang S., Jiang H. (2019). Vagus Nerve Stimulation Attenuates Acute Skeletal Muscle Injury Induced by Ischemia-Reperfusion in Rats. Oxid. Med. Cell. Longev..

[25] Greising S.M., Corona B.T., McGann C., Frankum J.K., Warren G.L. (2019). Therapeutic Approaches for Volumetric Muscle Loss Injury: A Systematic Review and Meta-Analysis. Tissue Eng. Part B Rev..

[26] Corona B.T., Henderson B.E., Ward C.L., Greising S.M. (2017). Contribution of minced muscle graft progenitor cells to muscle fiber formation after volumetric muscle loss injury in wild-type and immune deficient mice. Physiol. Rep..

[27] Grasman J.M., Do D.M., Page R.L., Pins G.D. (2015). Rapid release of growth factors regenerates force output in volumetric muscle loss injuries. Biomaterials.

[28] Quarta M., Cromie M., Chacon R., Blonigan J., Garcia V., Akimenko I., Hamer M., Paine P., Stok M., Shrager J.B. (2017). Bioengineered constructs combined with exercise enhance stem cell-mediated treatment of volumetric muscle loss. Nat. Commun..

[29] Mintz E.L., Passipieri J.A., Franklin I.R., Toscano V.M., Afferton E.C., Sharma P.R., Christ G.J. (2020). Long-Term Evaluation of Functional Outcomes Following Rat Volumetric Muscle Loss Injury and Repair. Tissue Eng. Part A.

[30] VanDusen K.W., Syverud B.C., Williams M.L., Lee J.D., Larkin L.M. (2014). Engineered skeletal muscle units for repair of volumetric muscle loss in the tibialis anterior muscle of a rat. Tissue Eng. Part A.

[31] Kasukonis B., Kim J., Brown L., Jones J., Ahmadi S., Washington T., Wolchok J. (2016). Codelivery of Infusion Decellularized Skeletal Muscle with Minced Muscle Autografts Improved Recovery from Volumetric Muscle Loss Injury in a Rat Model. Tissue Eng. Part A.

[32] Passipieri J.A., Hu X., Mintz E., Dienes J., Baker H.B., Wallace C.H., Blemker S.S., Christ G.J. (2019). In Silico and In Vivo Studies Detect Functional Repair Mechanisms in a Volumetric Muscle Loss Injury. Tissue Eng. Part A.

[33] Li M.T., Willett N.J., Uhrig B.A., Guldberg R.E., Warren G.L. (2014). Functional analysis of limb recovery following autograft treatment of volumetric muscle loss in the quadriceps femoris. J. Biomech..

[34] Anderson S.E., Han W.M., Srinivasa V., Mohiuddin M., Ruehle M.A., Moon J.Y., Shin E., San C.L., Emeterio M.E., Ogle E.A. (2019). Determination of a Critical Size Threshold for Volumetric Muscle Loss in the Mouse Quadriceps. Tissue Eng. Part C Methods.

[35] Ward C.L., Pollot B.E., Goldman S.M., Greising S.M., Wenke J.C., Corona B.T. (2016). Autologous Minced Muscle Grafts Improve Muscle Strength in a Porcine Model of Volumetric Muscle Loss Injury. J. Orthop. Trauma.

[36] Novakova S.S., Rodriguez B.L., Vega-Soto E.E., Nutter G.P., Armstrong R.E., Macpherson P.C.D., Larkin L.M. (2020). Repairing Volumetric Muscle Loss in the Ovine Peroneus Tertius Following a 3-Month Recovery. Tissue Eng. Part A.

[37] Sarrafian T.L., Bodine S.C., Murphy B., Grayson J.K., Stover S.M. (2018). Extracellular matrix scaffolds for treatment of large volume muscle injuries: A review. Vet. Surg..

[38] Matthias N., Hunt S.D., Wu J., Lo J., Callahan L.A.S., Li Y., Huard J., Darabi R. (2018). Volumetric muscle loss injury repair using in situ fibrin gel cast seeded with muscle-derived stem cells (MDSCs). Stem Cell Res..

[39] Page R.L., Malcuit C., Vilner L., Vojtic I., Shaw S., Hedblom E., Hu J., Pins G.D., Rolle M.W., Dominko T. (2011). Restoration of skeletal muscle defects with adult human cells delivered on fibrin microthreads. Tissue Eng. Part A.

[40] Tidball J.G. (2017). Regulation of muscle growth and regeneration by the immune system. Nat. Rev. Immunol..

[41] Goldman S.M., Corona B.T. (2017). Co-delivery of micronized urinary bladder matrix damps regenerative capacity of minced muscle grafts in the treatment of volumetric muscle loss injuries. PLoS ONE.

[42] Chen X.K., Walters T.J. (2013). Muscle-derived decellularised extracellular matrix improves functional recovery in a rat latissimus dorsi muscle defect model. J. Plast. Reconstr. Aesthet. Surg. JPRAS.

[43] Corona B.T., Machingal M.A., Criswell T., Vadhavkar M., Dannahower A.C., Bergman C., Zhao W., Christ G.J. (2012). Further development of a tissue engineered muscle repair construct in vitro for enhanced functional recovery following implantation in vivo in a murine model of volumetric muscle loss injury. Tissue Eng. Part A.

[44] Powell P.L., Roy R.R., Kanim P., Bello M.A., Edgerton V.R. (1984). Predictability of skeletal muscle tension from architectural determinations in guinea pig hindlimbs. J. Appl. Physiol. Respir. Environ. Exerc. Physiol..

[45] Mathewson M.A., Chapman M.A., Hentzen E.R., Friden J., Lieber R.L. (2012). Anatomical, architectural, and biochemical diversity of the murine forelimb muscles. J. Anat..

[46] Gilbert-Honick J., Iyer S.R., Somers S.M., Lovering R.M., Wagner K., Mao H.Q., Grayson W.L. (2018). Engineering functional and histological regeneration of vascularized skeletal muscle. Biomaterials.

[47] Nakayama K.H., Alcazar C., Yang G., Quarta M., Paine P., Doan L., Davies A., Rando T.A., Huang N.F. (2018). Rehabilitative exercise and spatially patterned nanofibrillar scaffolds enhance vascularization and innervation following volumetric muscle loss. NPJ Regen. Med..

[48] Sicari B.M., Rubin J.P., Dearth C.L., Wolf M.T., Ambrosio F., Boninger M., Turner N.J., Weber D.J., Simpson T.W., Wyse A. (2014). An acellular biologic scaffold promotes skeletal muscle formation in mice and humans with volumetric muscle loss. Sci. Transl. Med..

[49] Corona B.T., Ward C.L., Baker H.B., Walters T.J., Christ G.J. (2014). Implantation of in vitro tissue engineered muscle repair constructs and bladder acellular matrices partially restore in vivo skeletal muscle function in a rat model of volumetric muscle loss injury. Tissue Eng. Part A.

